# Combining balneotherapy and health promotion to promote active and healthy ageing: the Balaruc-MACVIA-LR^®^ approach

**DOI:** 10.1007/s40520-016-0596-4

**Published:** 2016-07-05

**Authors:** H. Blain, P. L. Bernard, G. Canovas, N. Raffort, H. Desfour, L. Soriteau, M. Noguès, T. Camuzat, J. Mercier, A. Dupeyron, I. Quéré, I. Laffont, C. Hérisson, H. Solimene, J. Bousquet

**Affiliations:** 1 Department of Geriatrics, Montpellier University Hospital, Montpellier, France; 2 EUROMOV. EA 2991, Euromov, University Montpellier, Montpellier, France; 3 Maire, Balaruc-Les-Bains, France; 4 Société Publique Locale d’Exploitation de Balaruc-les-Bains, Balaruc-Les-Bains, France; 5 Caisse Assurance Retraite et Santé Au Travail Languedoc-Roussillon (CARSAT-LR), Montpellier, France; 6 Montpellier, Région Languedoc-Roussillon-Midi-Pyrénées France; 7 PhyMedExp, INSERM U1046, CNRS UMR 9214, University of Montpellier, Montpellier, France; 8 Department of Physical and Medical Rehabilitation, Nîmes University Hospital, Nîmes, France; 9 Internal Medicine Department, Montpellier University Hospital, Montpellier, France; 10 Department of Physical and Medical Rehabilitation, Montpellier University Hospital, Montpellier, France; 11 School Of Medicine, State University of Milan, Milan, Italy; 12 WHO Collaborating Center For Traditional an Complementary Medicine, Milan, Italy; 13 FEMTEC (World Federation of Hydrotherapy and Climatotherapy), Milan, Italy; 14 University Hospital, Montpellier, France; 15 MACVIA-LR, Contre les MAladies Chroniques pour un VIeillissement Actif en Languedoc-Roussillon, European Innovation Partnership on Active and Healthy Ageing Reference Site, Montpellier, France; 16 INSERM, VIMA: Ageing and Chronic Diseases, Epidemiological and Public Health Approaches, U1168, Paris, France; 17 UVSQ, UMR-S 1168, Université Versailles St-Quentin-en-Yvelines, Versailles, France; 18 CHRU Montpellier, 34295 Montpellier, Cedex 5 France

**Keywords:** Balneotherapy, Climatotherapy, Falls, Education, Musculoskeletal disease

## Abstract

Scaling up and replication of successful innovative integrated care models for chronic diseases is one of the targets of the European Innovation Partnership on Active and Healthy Ageing (EIP on AHA). MACVIA-LR^®^ (MAladies Chroniques pour un VIeillissement Actif en Languedoc-Roussillon) is a Reference Site of the EIP on AHA. The main objective of MACVIA-LR^®^ is to develop innovative solutions in order to (1) improve the care of patients affected by chronic diseases, (2) reduce avoidable hospitalization and (3) scale up the innovation to regions of Europe. The MACVIA-LR^®^ project also aims to assess all possible aspects of medicine—including non-pharmacologic approaches—in order to maintain health and prevent chronic diseases. These approaches include hydrotherapy and balneotherapy which can be of great importance if health promotion strategies are considered. Balneotherapy at Balaruc-les-Bains focusses on musculoskeletal diseases and chronic venous insufficiency of the lower limbs. Each year, over 46,000 people attend an 18-day course related to a new falls prevention initiative combining balneotherapy and education. On arrival, each person receives a flyer providing information on the risk of fall and, depending on this risk, a course is proposed combining education and physical activity. A pilot study assesses the impact of the course 6 and 12 months later. This health promotion strategy for active and healthy ageing follows the FEMTEC (World Federation of Hydrotherapy and Climatotherapy) concept.

## Introduction

The novel trend for the management of chronic diseases is evolving and is leading towards integrative approaches that include health promotion and non-pharmacologic therapies. Active and Healthy Ageing (AHA) is being promoted through these approaches. The European Innovation Partnership on AHA (EIP on AHA) is deployed in 3 areas and 6 action plans including the scaling up and replication of successful innovative integrated care models for chronic diseases amongst older patients [1].

The Région Languedoc-Roussillon is the umbrella organization for an interconnected and integrated project on AHA which covers the 3 pillars of the EIP on AHA [2]. All subactivities are included in MACVIA-LR^®^ (MAladies Chroniques pour un VIeillissement Actif en Languedoc-Roussillon), one of the Reference Sites of the EIP on AHA, built around chronic diseases, ageing and disability. The MACVIA-LR^®^ framework has the vision that the prevention and management of NCDs are essential for AHA promotion and for reducing disability. The main objective of MACVIA-LR^®^ is to develop innovative solutions in order to (1) improve the care of patients affected by NCDs, (2) reduce avoidable hospitalization and (3) scale up the innovation to regions of Europe. One of the strengths of MACVIA-LR is the sound link between hydrotherapy/balneotherapy health resorts and the teaching hospitals of the Region.

The MACVIA-LR^®^ project also aims to incorporate all possible aspects of medicine—including non-pharmacologic approaches—in order to maintain health and prevent NCDs. These approaches include hydrotherapy and balneotherapy which can be of great importance if health promotion strategies are considered and if the FEMTEC concept is followed. This concept was developed by Santuari and Solimene who propose to focus on prevention and health promotion rather than the concept of cure [3].

## Balneotherapy in musculoskeletal diseases and phlebology

The use of water for various treatments (hydrotherapy, spa or balneotherapy) is probably as old as mankind. Balneotherapy is one of the basic methods of treatment widely used in the system of natural medicine. However, Health Resort Medicine, Balneology, Medical Hydrology and Climatology are not fully recognized as independent medical specialities at a global international level [4]. Although large studies are lacking, balneotherapy has a scientific evidence-based effect on various systems of the body [5] and in musculoskeletal diseases in particular [6–9]. It is used in subjects with a wide variety of disease severity and, often, in subjects who have a normal or subnormal quality-of-life without major impairment. In these subjects, balneotherapy promotes AHA. In patients with a severe disease and/or disability, balneotherapy helps to improve physical function or to relieve pain [10]. It can therefore impact all body systems including depression (inconsistent results), other mental health problems and quality-of-life [9]. In the vast majority of places, treatment is centred only around the benefits of balneotherapy although it has been recommended to consider its combination with education [11]. The Secretary General of FEMTEC, a member of the Global Alliance against Chronic Respiratory Diseases (World Health Organisation), recently wrote a statement considering balneotherapy as an important alternative medicine to promote AHA.

## Balneotherapy at Balaruc-les-Bains

**Fig. 1:**
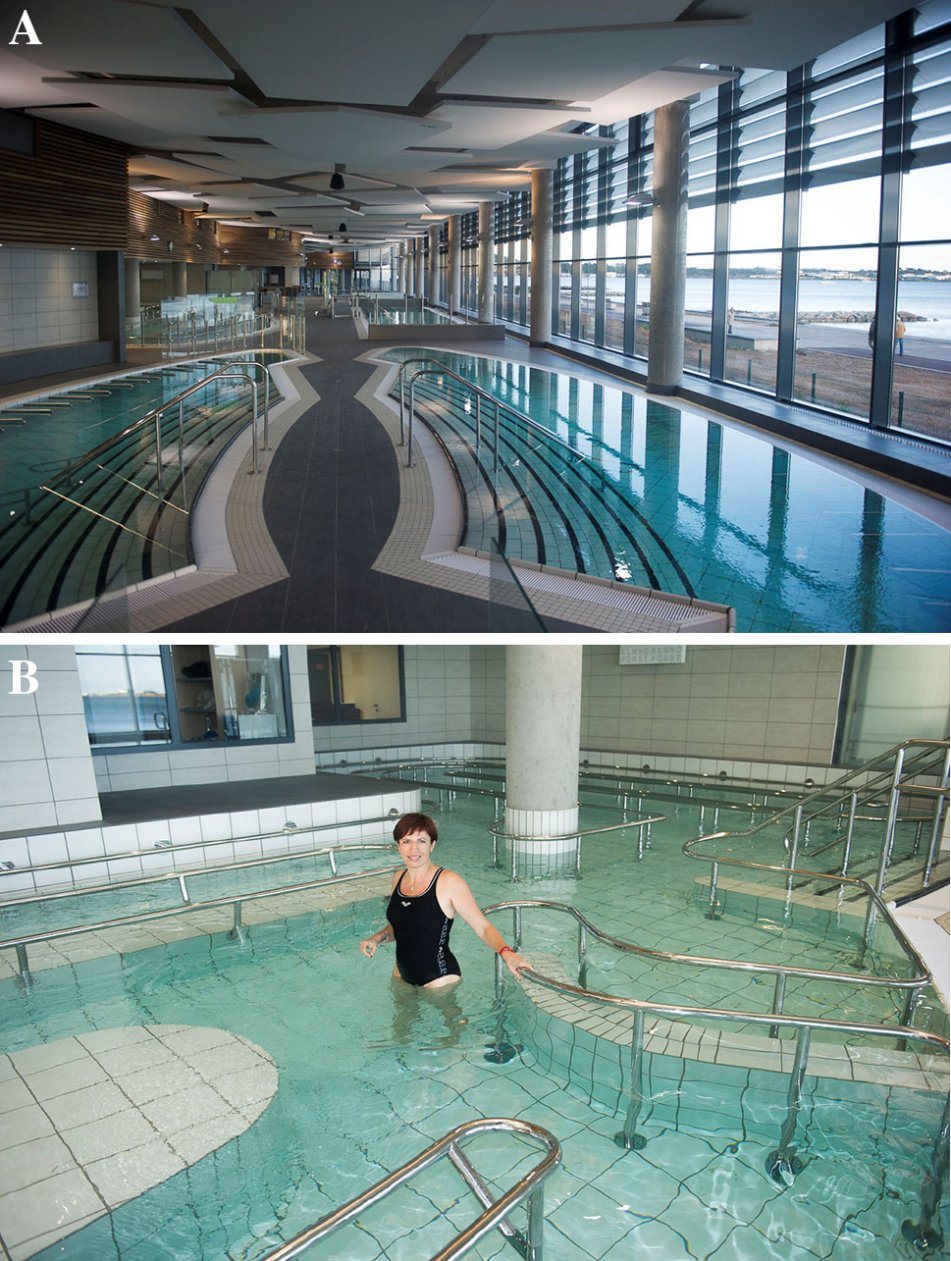
**a** View of the interior facilities related to falls prevention, b the walking corridor [14, 15]

Balneotherapy at Balaruc-les-Bains encompasses the objectives of the Ottawa Charter (WHO 1986) whose aim is to “enable people to increase control over and improve their health”. The spa treatment lasts for 18 days and is an opportunity to promote health and all the good habits that go with it. In order to achieve the objectives, 400 m^2^ are available to promote the health and education of the patients (Fig. 1a, b). The Balaruc-les-Bains health resort is committed to developing the capabilities of its patients with regards to health. Its aim is to improve their knowledge and to convey useful everyday skills in order to ensure a greater control over their own health.

The climatic health resort of Balaruc-les-Bains (in the Hérault department) was scientifically recognized in the 16th century for the quality of its thermal water and for its dual therapeutic orientation in rheumatology and phlebology. The centre welcomes over 46,000 spa users per year. In 2014, Balaruc-les-Bains became the most frequently visited Spa in France. This represents more than a third of the increase in French balneotherapy over the same period.

Balaruc-les-Bains focusses on musculoskeletal diseases and chronic venous insufficiency of the lower limbs [12]. A falls prevention programme exists in MACVIA-LR [13], and the development of the falls screening programme for subjects with musculosketal diseases is important in order to propose simple but efficient measures to reduce falls (Fig. 1).

The new centre is spread over 16,000 m^2^ and includes 5 pools for group therapy in rheumatology and phlebology. It also provides individual hydrotherapy treatment of which 4 sectors specialize in the application of thermal mud.

The Balaruc-les-Bains Spa is innovative in several ways. In rheumatology, a new bed has been specially designed for the application of thermal mud. This device applies peloid mud, an organic mineral complex made up of a smooth liquid mixture of Balaruc-les-Bains clay and thermal water. This mixture is applied directly around joint and spine areas depending on the medical prescription. The significant amount of thermal water contained in the peloid mud increases therapeutic efficacy by associating the richness of the oligo elements and the benefits of a constant heat (42°) throughout the treatment.

In the phlebology sector, a walking corridor has been specially designed to alleviate circulatory disorders in the lower limbs and to increase joint mobility and balance. Walking on a carpet of tiny air bubbles stimulates proprioception and microcirculation. It also increases venous return by the calf muscle pump which accelerates when walking against the current. Balance therapy using an inclined surface (uphill and downhill) of pebbles or tiles—depending on the pathology—enables stimulation of the plantar arch and venous pump, relaxation of the ankles as well as an improved physical perception of the ground.

## Falls prevention combining balneotherapy and education

The new centre combines the most effective balneotherapy methods with therapeutic education. This combination will be used to promote AHA and will be tested to confirm its benefits. The experiment will be scaled up at the EU level using the EIP on AHA.

A new falls prevention initiative has been set up and a pilot study initiated. On arrival, each person receives a flyer containing information on the risk of fall. A questionnaire is also distributed in which the STEADI algorithm (Stopping Elderly Accidents, Deaths and Injuries; Centers for diseases Control and Prevention) (http://www.cdc.gov/steadi/) [16] is used to assess the risk of falling. This study is in place and the methodology was developed by members of the University of Montpellier. More specifically, three questions are included in the first examination (Table 1).

**Table 1:** Screening questions

Have you fallen in the past year?
Do you feel unsteady when standing or walking?
Do you worry about falling?

Those who reply “yes” to any of these key screening questions are considered at increased risk of falling, and further assessment is then performed (Table 2).

**Table 2:** Assessment of risk of falling and physical performance

Gait speed [17]
Timed Up & Go [18]
30 s Chair Stand [19]
One leg stand time (eyes open and eyes closed) [20]
Measure of fear of falling [21]

A multicomponent physical activity programme is then proposed. It includes exercises that have been shown to reduce the risk of fall in people at moderate risk, associated with an educational health programme [14, 15, 22, 23]. The physical activity programme includes balance exercises, along with exercises to improve muscle strength and articular mobility, once a day, 40 min/day, 5 days/week, for the whole stay (3 weeks). The educational intervention is designed to increase knowledge relating to falls prevention. The programme is based on those used in previous studies [24, 25].

On departure, the parameters measured at baseline are reassessed and a list of certified organizations (taking home location into account) is provided to promote further continuation of the physical activity programme. A letter is sent to the patient’s GP informing him/her of the risk of falling. The GP is also informed of the programme performed by the patient during his/her stay as well as the advice given. (http://www.cdc.gov/steadi/; http://www.cdc.gov/steadi/pdf/algorithm_2015-04-a.pdf).

A follow-up using a standardized questionnaire is organized by telephone at 1, 3, 6 and 12 months to check if the programme has been pursued and to assess its effect on the number of falls, fear of falling, activities in daily living, and quality-of-life.

## Conclusions

Resorts such as Balaruc-les-Bains receive older patients with chronic venous insufficiency and musculoskeletal diseases. Considering that a third of the people aged 65 or older in the general population experience at least one fall every year, a large number of patients handled in the resort are at moderate or high risk of falling. The 18-day stay of the patients in the resort enables a multicomponent physical activity programme associated with an educational intervention. The experience of Balaruc-les-Bains is a MACVIA-LR^®^ initiative. It can be further scaled up to other health resorts in Europe and beyond with FEMTEC in order for Health spa centres to play a significant role in preventive medicine and to be regarded as an important component of the overall “health market”.

## Abbreviations

AHA: Active and healthy ageing
EIP: European Innovation Partnership
FEMTEC: World federation of hydrotherapy
MACVIA-LR^®^: MAladies Chroniques pour un VIeillissement Actif en Languedoc-Roussillon
NCDs: Non-communicable diseases
